# Symptom Trajectories in Patients With Anxiety Disorders During Transdiagnostic Cognitive Behavioral Therapy

**DOI:** 10.1155/da/2743962

**Published:** 2026-07-21

**Authors:** Laura E. Meine, Carmen Schaeuffele, Dominique Recher, Miriam Müller-Bardorff, Christina Paersch, Ava Schulz, Isaac Galatzer-Levy, Birgit Kleim

**Affiliations:** ^1^ Experimental Psychopathology and Psychotherapy, Department of Psychology, University of Zurich, Zurich, Switzerland, uzh.ch; ^2^ Department of Adult Psychiatry and Psychotherapy, Psychiatric University Clinic Zurich and University of Zurich, Zurich, Switzerland; ^3^ Department of Education and Psychology, Freie Universität Berlin, Berlin, Germany, fu-berlin.de; ^4^ Department of Psychiatry, New York University Grossman School of Medicine, New York, New York, USA, med.nyu.edu

**Keywords:** anxiety disorders, latent class growth analysis, transdiagnostic cognitive behavioral therapy, Unified Protocol

## Abstract

Growing research confirms the efficacy of transdiagnostic cognitive behavioral psychotherapy, that is treatments applicable across multiple diagnoses within a disorder class or spectrum, by examining group‐level changes from pre‐ to posttreatment. However, less is known about interindividual differences and dynamic changes during therapy. Understanding different symptom trajectories and their associations with treatment outcome would allow early detection of nonresponders and treatment adjustments. Using data from the treatment group of a randomized controlled trial, we aimed to investigate trajectories in transdiagnostic cognitive behavioral therapy (CBT) with the Unified Protocol (UP) in patients with anxiety disorders. Patients reported on demographics, clinical history, coping strategies, treatment motivation, and expectations before starting transdiagnostic CBT. Participants provided symptom ratings across 16 sessions of the UP as well as at posttreatment, 6‐, and 12‐month follow‐up (*N* = 64, 71.9% female, age: *M* = 32.67, *SD* = 11.91). Using latent class growth analysis (LCGA), we identified symptom trajectory classes and tested whether these could be predicted from baseline data. We also examined associations between classes and treatment outcome, using multilevel modeling and reliable change indices (RCIs). LCGA revealed four distinct trajectory classes: low severity—improved (*n* = 21, 32.81%), high severity—improved (*n* = 19, 29.69%), high severity—stagnant (*n* = 12, 18.75%), and low severity—worsened (*n* = 12, 18.75%). Only the number of comorbidities emerged as a significant predictor of class, with patients in the high severity—stagnant group having more comorbidities. They also showed higher symptom severity at baseline, posttreatment, and follow‐ups compared to other classes, despite clear improvement. Latent classes of symptom changes during transdiagnostic CBT can be distinguished and important pretreatment factors identified, informing treatment selection and personalization. Particularly patients with high initial burden and a higher number of comorbidities may require monitoring and adaptive treatment strategies, which should be explored in future studies.

**Trial Registration:** ClinicalTrials.gov identifier: NCT03945617

## 1. Introduction

Anxiety disorders are the most prevalent group of mental disorders [1]. This includes patients with complaints in the domain of social anxiety disorder, generalized anxiety disorder, panic disorder, agoraphobia, and specific phobias. Anxiety disorders tend to co‐occur [2]. Transdiagnostic cognitive behavioral therapy (CBT) has the potential to treat anxiety disorders effectively and, in the presence of more than one anxiety disorder at the time, simultaneously [3]. A recent meta‐analysis found transdiagnostic CBT to be effective in reducing anxiety and depression with medium‐to‐large effects [4]. One example of a transdiagnostic CBT approach is the Unified Protocol (UP), which promotes an approach‐oriented stance towards emotions [5].

While the UP’s effects are promising, studies have mainly investigated pre–post changes in symptoms rather than considering trajectories over therapy [6]. Such group‐level changes from pre to post may mask variability in treatment effects and dynamic changes over the course of treatment. Disaggregating this and uncovering different trajectories of changes over treatment allows for a more nuanced understanding of the timing and interindividual differences in treatment response.

Symptom trajectories have been investigated in different disorders and in different treatments. As these treatments are heterogeneous in length, session frequency (e.g., weekly vs. biweekly therapy frequency: [7]), setting (for an example in the internet‐based setting: [8]), and breadth of application, the exact number of treatment trajectory classes varies between studies. However, three classes have often been identified: early, delayed, or no change (e.g., [7, 9–11]). Only a small proportion of patients tends to worsen over time. The majority of studies investigated symptom trajectories in a specific disorder, for example, depression (e.g., [11]) or panic disorder (e.g., [10]). Identifying who benefits from treatment and which factors promote beneficial outcome cases has important clinical implications: recognizing early on in treatment who benefits may help to reduce treatment failure, as it may allow one to suggest alternative treatments or show avenues for personalization, like prioritizing certain treatment modules or intensifying treatment frequency (e.g., [12]).

Overall, little is known about trajectories of symptom changes in transdiagnostic samples during transdiagnostic treatment. A recent study investigated symptom trajectories in transdiagnostic behavior therapy in a mixed sample of patients with depression, posttraumatic stress disorder (PTSD), and panic disorder [13]. They identified three groups of response patterns: high responders, moderate responders, and low responders. In panic disorder and PTSD patients, higher baseline severity as well as high behavioral avoidance and low homework compliance were predictive of nonresponse. For the UP, trajectories were investigated for the youth population [14]. They identified three classes: two steady improvement classes with moderate and low baseline severity, as well as a high rapid improvement class with high baseline severity. The majority of youths (64%) showed moderate baseline severity with steady improvement. In a sample of mixed common mental health disorders undergoing psychological therapy, Saunders and colleagues [15] identified that distinct trajectories of treatment response were identifiable by session three. For anxiety, they identified five classes of response patterns: On track cases seemed to be associated with lower baseline severity, better social functioning, and a lower incidence of phobic anxiety.

While trajectories have mostly been linked to baseline symptom severity, testing associations with transdiagnostic processes such as approach and avoidant coping or early treatment factors such as motivation, goals, and expectations may help uncover the mechanisms giving rise to interindividual differences in symptom improvement over therapy. Coping strategies, and in particular the balance between active and avoidant coping, may be especially relevant for anxiety disorders, where avoidance is considered a core mechanism maintaining symptoms and is directly targeted by the UP through its focus on reducing emotional avoidance and fostering an approach‐oriented reaction to emotions. Similarly, focusing on early treatment factors may be informative. For instance, while higher expectations are generally associated with better outcomes posttreatment (e.g., [16, 17]), examining their relation to trajectories may yield more nuanced insights: in one study of depressed patients, higher expectations predicted a delayed response to treatment [18]. Patients who begin therapy with high hopes, expecting a quick recovery, may experience (temporary) deterioration in functioning when symptom improvements do not materialize immediately. In addition, factors like coping, expectation, motivations, and goals—in contrast to demographic or clinical baseline factors—may be a target for early intervention to set patients up for treatment.

To help close the above‐mentioned gaps in the literature, aims for this study were threefold: The primary aim was to investigate interindividual differences in symptom trajectories in patients with mixed anxiety disorders during transdiagnostic CBT with the UP. The second goal was to gain a better understanding of what characterizes these classes. Proxy variables that are associated with specific classes of treatment trajectories may help identify patients at risk of treatment failure even earlier, that is, at baseline or early in treatment [19]. We investigated whether emerging latent symptom change classes are distinguished by relevant baseline, including sociodemographic and clinical and transdiagnostic markers, and early treatment factors, including expectations, alliances, and goals. The third aim was to understand whether classes of symptom trajectories are associated with outcomes directly after therapy and at 6‐ and 12‐month follow‐up to corroborate symptom trajectories and investigate maintenance of treatment gains.

## 2. Materials and Methods

This is a secondary analysis of the OPTIMAX randomized controlled trial investigating the UP versus waitlist [20]. For this analysis, only data from the treatment group were used: *N* = 71 patients with a primary anxiety disorder underwent an average of 16 sessions of transdiagnostic CBT according to the UP and reported the extent of their anxiety symptoms in each session using the Overall Anxiety Severity and Impairment Scale (OASIS; [21]). Although waitlist participants received the same treatment after a 16‐week waiting period, session‐by‐session symptom ratings were not collected with the same density for this group once treatment began. This reflected an ethical decision, in line with the study’s ethics approval, not to require further routine assessments from participants who had already completed an extensive assessment battery during the waiting period and were not obligated to take on additional monitoring once finally receiving treatment. Consequently, this subgroup could not be included in the present trajectory analyses, which are based on the immediate‐treatment group only. The trial was registered on ClinicalTrials.gov and details on study procedures can be found in the study protocol [20]. The study was approved by the cantonal ethics committee of Zurich (BASEC Number 2017–01443) and carried out in accordance with the Declaration of Helsinki and the Good Clinical Practice (GCP) guideline. We adhere to the Strengthening the Reporting of Observational Studies in Epidemiology (STROBE) guidelines (see Supporting Information 1 for the checklist).

### 2.1. Participants

We recruited participants through different channels, including via social media and the study website, mailing lists, newspaper articles, general practitioners’ offices, and anxiety self‐help groups in Zürich. We determined eligibility with a telephone screening and clinical interview according to DSM‐5. After giving written informed consent, participants were eligible if they presented with a primary diagnosis of an anxiety disorder (i.e., panic disorder, agoraphobia, social anxiety disorder, generalized anxiety disorder, anxiety disorder not otherwise specified, adjustment disorder with anxiety, adjustment disorder with mixed anxiety and depressed mood), were between 18 and 65 years of age, were fluent in German, were currently not in psychotherapy, and showed a stable dose of medication over the previous 3 months if medicated. We excluded participants if they had a potentially interfering medical contraindication (e.g., cardiovascular diseases and autoimmune diseases), a history of schizophrenia, psychosis, bipolar disorder, or A or B personality disorder, current substance dependence or abuse (except for nicotine), or showed suicidal ideation. After exclusion of dropouts, the final sample comprised *N* = 64 patients (71.9% female, age: *M* = 32.67, *SD* = 11.91, range: 18−60 years). Data were available from *n* = 62 at posttreatment and *n* = 60 at both follow‐ups.

### 2.2. Treatment

Treatment consisted of 16 sessions of individual UP on average. The UP is a transdiagnostic CBT intervention for anxiety disorders [5]. The UP aims at promoting a flexible and adaptive manner in which patients relate to and regulate their emotions. We applied the UP modules in standard order: (1) Introduction and motivation enhancement, (2) identifying and understanding emotions, (3) emotional awareness training, (4) cognitive flexibility training, (5) emotional avoidance and emotion‐driven behaviors, (6) awareness and tolerance of bodily sensations, (7) interoceptive and situation‐based exposures, and (8) relapse prevention. The UP was provided by four study therapists with training in the UP and several years of clinical experience. To ensure treatment adherence, therapy was supervised by a senior clinician.

### 2.3. Measures

#### 2.3.1. Session‐to‐Session Symptom Change Measure

As a session‐by‐session measure of anxiety, we applied the OASIS, a five‐item self‐report scale, which has shown excellent psychometric properties across anxiety disorders [21]. OASIS ratings were used to investigate interindividual differences in symptom trajectories.

#### 2.3.2. Characterizations of Symptom Trajectory Classes

We used a range of predictors including symptoms, sociodemographic information, and more general therapeutic mechanisms to characterize symptom trajectory classes. In regard to symptoms, we investigated the primary diagnosis according to the Mini International Neuropsychiatric Interview (MINI; [22]), number of comorbid diagnoses, anxiety clinician‐report (Hamilton Anxiety Scale; HAMA; [23]), anxiety self‐report (Beck Anxiety Inventory; BAI; [24]), and depression self‐report (Beck Depression Inventory; BDI‐II, [25]) at baseline. For sociodemographic information, we explored age, biological sex, education, medication, and a marker for chronicity, that is, past psychotherapy. For therapeutic mechanisms, we explored coping, expectations, motivation, and the working alliance. We used the Patient Questionnaire on Therapy Expectation and Evaluation (PATHEV; [26]) to assess expectations. The PATHEV measures the expectation‐related constructs hope of improvement, fear of change, and suitability, with 11 items on a 5‐point scale. The “Fragebogen zur Therapiemotivation (FPTM)” was used to assess therapy motivation with its subscales denial of need for psychological help, hope, initiative, and knowledge, with 16 items on a 4‐point scale [27]. We used the short revised Working Alliance Inventory (WAI‐SR) to assess the working alliance, with the subscales bond, task, and goal, with 12 items on a 5‐point scale [28]. We used the Brief COPE [29, 30] to assess coping/emotion regulation strategies. The brief COPE captures a range of adaptive and maladaptive coping responses across 14 conceptual subscales with 2 items each, rated on a 4‐point scale. We focused on the active and avoidant coping subscales, as these are conceptually most closely aligned with the UP’s core treatment target of reducing emotion‐motivated avoidance, which is implicated in the maintenance and exacerbation of anxiety disorders.

#### 2.3.3. Outcome Measures

We included clinician‐ and self‐rated anxiety as outcomes. For the clinician rating, we used the HAMA at pre‐ and posttreatment. Clinicians rated 14 items reflecting psychological and somatic aspects of anxiety on a 5‐point scale. For the self‐report rating, we used the BAI at pre‐ and posttreatment as well as at 6‐ and 12‐month follow‐up. Patients rated 21 questions related to their anxiety on a 4‐point scale. Additionally, we included patients’ depression self‐report (BDI‐II) at posttreatment.

### 2.4. Data Analysis

Data were analyzed using R (Version 4.3.2). We examined the number of sessions per patient (*M* = 16.5, *SD* = 2.35, range = 7–21), which averaged about 16 in accordance with the recommended number of sessions for the UP. Symptom reports per session were very regular, with only a few participants missing a few reports (see Supporting Information 2: Figure S1). A total of 39 (60.9%) patients had up to 18 sessions, and six (9.4%) completed up to 21 sessions. We based the identification of trajectory classes on data from 16 sessions, including all patients. We conducted a sensitivity analysis for trajectory classes based on data from 18 sessions and examined the trajectories of patients with more than 16 sessions.

#### 2.4.1. Latent Class Growth Analysis (LCGA)

First, we established that our data showed sufficient heterogeneity to warrant a LCGA. To do so, we set up a linear mixed‐effects model predicting symptom severity by session (measured with the OASIS) and including random intercepts for patients. A comparison with a model including random slopes revealed a significantly better fit (*χ*2(2) = 121, *p* <  0.001), indicating that LCGA was appropriate for our data. We opted for LCGA over growth mixture modeling (GMM) given sample size constraints on model complexity and convergence stability. Unlike GMM, where within‐class variance in intercepts and slopes is freely estimated, LCGA fixes these parameters to zero, capturing individual deviation from the class‐specific mean trajectory solely through residual variance. Thus, using this more parsimonious method, we subsequently estimated models with one to five symptom trajectory classes, fitted with 100 random starts to avoid local maxima, and 10 iterations. To choose the best solution, we examined commonly used fit indices [31]. A lower Akaike information criterion (AIC), Bayesian information criterion (BIC), sample‐size‐adjusted BIC (SABIC), and integrated completed likelihood (ICL) reflect better model fit. High classification accuracy is indicated by entropy values close to one. We conducted adjusted Lo‐Mendell‐Rubin Likelihood Ratio Tests (LMR‐LRTs) to check whether adding a class significantly improved model fit. Moreover, we considered the number of participants in the smallest class (aiming for at least 10) and plotted the resulting class trajectories to assess theoretical interpretability. LCGA was conducted using the package “lcmm” [32].

#### 2.4.2. Analysis of Symptom Trajectory Classes

We examined class differences in sociodemographic and baseline characteristics as well as early treatment factors using descriptive statistics. We then conducted multinomial logistic regression, predicting latent class membership by predefined variables on which classes appeared to differ, testing statistical significance. We refrained from including all variables in our model due to sample size constraints.

To investigate whether identified latent symptom trajectory classes differed in therapy outcome at posttreatment and follow‐ups, we provide descriptive statistics of BAI and HAMA scores at pre‐, post‐, and follow‐ups, separately for the four classes. To test statistical significance, we set up a linear mixed‐effects model, predicting symptoms by time, class, and the interaction of time and class, including by‐participant random intercepts. We used the “lme4” package [33] and conducted post hoc comparisons with the “emmeans” package [34]. Furthermore, we calculated reliable change indices (RCIs) to gauge the reliability and clinical relevance of symptom improvement from pre‐ to posttreatment according to Jacobson and Truax [35], using the “JTRCI” package [36]. This method assesses (1) whether improvements are reliable and not due to measurement error or natural variability and (2) whether improvements are clinically meaningful by comparing with established norm values or clinical cutoffs. We focused on RCIs for self‐reported symptoms based on BAI scores and compared them between latent trajectory classes. We specified a retest reliability of 0.65 [37] and included normative (*M* = 3.4, *SD* = 5.8) and dysfunctional means and SDs (*M* = 23.6, *SD* = 12) based on scores reported in the German BAI manual [38]. For clinician‐rated symptoms assessed with the HAMA, no norm data was available, but the instruction to the German HAMA contains means and SDs for different anxiety populations assessed with the English‐speaking HAMA. To compare categories with the BAI, we opted to calculate the RCI using means and SDs from GAD patients, as reported in [39]. Results are available in Supporting Information 2: Table S5. In addition, we provide RCIs calculated without norm values for BAI and HAMA in Supporting Information 2: Table S6, as well as Jacobson‐Truax plots for all results in Supporting Information 2: Figures S5–S8. These scatter plots show pre‐ vs. posttreatment symptom scores. Lines indicate thresholds for reliable change and normative functioning, distinguishing individuals as “deteriorated,” “unchanged,” “improved,” “nonreliably recovered,” or “recovered.”

A preliminary version of this manuscript was previously posted as a preprint [40] and is identical in content, differing only in minor formatting.

## 3. Results

### 3.1. Symptom Trajectory Classes

All models converged, and fit statistics indicated better fit with increasing numbers of classes, suggesting the five‐class solution (Table 1; also see Supporting Information 2: Table S1 for all class percentages). Visual inspection of the class trajectories revealed a similar pattern for two classes, both starting with comparatively higher symptom scores and improving over the course of treatment (see Supporting Information 2: Figure S2). For better theoretical interpretability and bigger class sizes, we selected the similarly well‐fitting four‐class solution as the final model. Average latent class probabilities, which indicate that patients were correctly assigned to the class to which they are most likely to belong, ranged between 0.93 and 0.98 (see Supporting Information 2: Table S2 for all probabilities).

**Table 1:** Goodness of fit statistics for the 1–5 class model of symptom trajectories.

Classes	Loglik	AIC	BIC	SABIC	Entropy	ICL	LMR‐LRT
1	−2677	5360	5366	5357	1.0000	5366	—
2	−2480	4973	4986	4967	0.9260	4989	<0.001
3	−2409	4837	4856	4828	0.9452	4859	<0.001
**4**	**−2381**	**4786**	**4812**	**4774**	**0.9231**	**4819**	**<0.001**
5	−2350	4730	4762	4715	0.9421	4766	<0.001

*Note*: Fit statistics for the selected model were bolded. ICL, integrated completed likelihood (we report “ICL2” values output by “lcmm” which are calculated with greater emphasis on parsimony); LMR‐LRT, adjusted Lo‐Mendell‐Rubin likelihood ratio test.

Abbreviations: AIC, Akaike information criterion; BIC, Bayesian information criterion; SABIC, sample‐size adjusted BIC.

**Figure 1:**
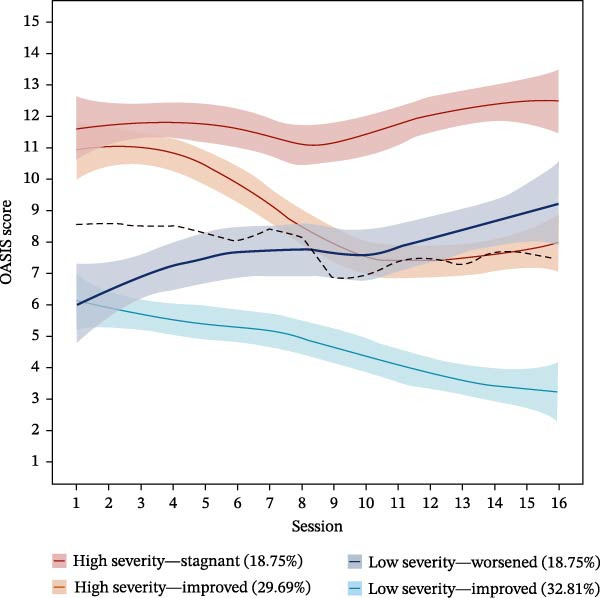
Trajectories of anxiety symptom severity according to the four‐class model. The dashed black line depicts the sample mean.

As the median number of sessions was 17, we investigated the trajectories over 16 sessions. The largest class in the four‐class model (Figure 1) comprised 32.81% of the sample (*n* = 21) and reflects patients with low initial symptom severity (intercept = 6.24 ± 0.35, *p* <  0.001) which reduced continuously over the course of treatment (slope = −0.22 ± 0.04, *p* <  0.001). The second‐largest class (29.69%, *n* = 19) had higher symptom severity at the start of treatment (intercept = 11.82 ± 0.49, *p* <  0.001), and showed marked improvement after a few sessions, then stabilizing at a moderate symptom level (slope = −0.32 ± 0.05, *p* <  0.001). The remaining two classes both comprised 18.75% of the sample (*n* = 12) and followed less favorable trajectories with one showing consistently high symptom severity with no significant change over the course of therapy (intercept = 11.73 ± 0.46, *p* < 0.001; slope = 0.09 ± 0.05, *p* = 0.083) and the other exhibiting a continuous significant increase in symptoms (intercept = 6.31 ± 0.69, *p* <  0.001; slope = 0.19 ± 0.07, *p* =  0.007).

A sensitivity analysis based on data from 18 therapy sessions revealed highly comparable results (see Supporting Information 2: Table S3 and Supporting Information 2: Figure S4), with 53 (83%) patients assigned to the same class as in the model based on data from 16 sessions. Of the 11 (17%) participants who switched classes, nearly half switched between the middle classes (i.e., from high severity—improved to low severity—worsened or vice versa).

### 3.2. Symptom Trajectory Classes and Baseline Characteristics

Descriptive comparisons of baseline characteristics revealed mixed findings on differences between latent trajectory classes; however, the high severity—stagnant class stood out with regard to some indices (see Table 2). More patients in this group were men, had a lower level of education, a higher number of comorbidities, and reported taking medication. To ascertain whether these differences were statistically significant, we set up a multinomial logistic regression analysis, predicting latent class membership by sex, level of education, number of comorbidities, and medication status. To simplify, we binarized education into lower (mandatory school years, apprenticeship, and high school) and higher (university of applied sciences, university) levels. The high‐severity stagnant class was set as the reference category and compared to each of the other classes. The regression model did not show good fit (*χ* ^2^ = 17, *p* = 0.149, McFadden *R* ^2^ = 0.10), correctly classifying only 40% of cases. This likely reflects the small sample size, and results should be interpreted with caution. Only the number of comorbidities was found to predict latent class membership (see detailed results in Supporting Information 2: Table S7) with patients in the high severity—stagnant class having significantly more comorbidities than those in the low severity—improved class (OR = 0.50, 95% CI = [0.26, 0.98], *p* = 0.045) and marginally more than those in the low severity—worsened class (OR = 0.43, 95% CI = [0.18, 1.01], *p* = 0.053). Compared to the high severity—stagnant class, there were fewer men in the other classes; however, effects did not reach statistical significance.

**Table 2:** Baseline characteristics by symptom trajectory class.

Characteristic	High severity—improved *n* = 19^1^	High severity—stagnant *n* = 12^1^	Low severity—improved *n* = 21^1^	Low severity—worsened *n* = 12^1^
Age	31 (10.44)	36 (14.62)	36 (12.65)	27 (6.78)
Sex				
Female	16 (84.21%)	6 (50.00%)	13 (61.90%)	11 (91.67%)
Male	3 (15.79%)	6 (50.00%)	8 (38.10%)	1 (8.33%)
Education				
9 mandatory school years	0 (0.00%)	1 (8.33%)	0 (0.00%)	0 (0.00%)
Apprenticeship	3 (15.79%)	5 (41.67%)	8 (38.10%)	2 (16.67%)
High school	6 (31.58%)	2 (16.67%)	6 (28.57%)	4 (33.33%)
University	8 (42.11%)	4 (33.33%)	5 (23.81%)	4 (33.33%)
University of applied sciences	2 (10.53%)	0 (0.00%)	2 (9.52%)	2 (16.67%)
Primary diagnosis				
AdjD	0 (0.00%)	0 (0.00%)	0 (0.00%)	1 (8.33%)
GAD	6 (31.58%)	4 (33.33%)	3 (14.29%)	1 (8.33%)
PD	1 (5.26%)	0 (0.00%)	0 (0.00%)	0 (0.00%)
PD/AG	0 (0.00%)	3 (25.00%)	9 (42.86%)	1 (8.33%)
SAD	10 (52.63%)	5 (41.67%)	7 (33.33%)	9 (75.00%)
SP	2 (10.53%)	0 (0.00%)	2 (9.52%)	0 (0.00%)
Number of comorbidities				
0	6 (31.58%)	2 (16.67%)	5 (23.81%)	3 (25.00%)
1	3 (15.79%)	3 (25.00%)	10 (47.62%)	7 (58.33%)
2	7 (36.84%)	2 (16.67%)	5 (23.81%)	2 (16.67%)
3	2 (10.53%)	3 (25.00%)	0 (0.00%)	0 (0.00%)
4	1 (5.26%)	1 (8.33%)	1 (4.76%)	0 (0.00%)
5	0 (0.00%)	1 (8.33%)	0 (0.00%)	0 (0.00%)
Medication	6 (33.33%)	6 (50.00%)	7 (33.33%)	5 (41.67%)
Past psychotherapy	11 (61.11%)	8 (66.67%)	13 (61.90%)	8 (66.67%)
PATHEV fear	2 (0.89)	2 (0.61)	1 (0.51)	2 (0.81)
PATHEV hope	4 (0.50)	3 (0.61)	4 (0.54)	3 (0.84)
PATHEV suitability	4 (0.47)	4 (0.53)	4 (0.64)	4 (0.36)
FPTM hope	13 (1.69)	12 (2.35)	13 (2.15)	12 (2.11)
FPTM initiative	9 (2.27)	8 (1.44)	9 (2.53)	9 (1.11)
FPTM knowledge	9 (2.59)	9 (2.60)	9 (2.43)	9 (2.18)
FPTM denial	14 (2.00)	14 (2.81)	13 (2.56)	13 (3.92)
WAI goal	18 (2.17)	17 (2.63)	18 (2.14)	17 (2.12)
WAI task	14 (3.31)	14 (3.28)	14 (3.14)	12 (2.35)
WAI bond	17 (2.04)	17 (3.23)	17 (2.58)	18 (2.10)
Brief COPE active	3 (0.62)	3 (0.77)	3 (0.80)	2 (0.67)
Brief COPE avoidant	2 (0.47)	2 (0.36)	2 (0.53)	2 (0.78)

*Note:* PATHEV, Measurement of therapy expectation and therapy evaluation of patients; FPTM, Questionnaire for the assessment of psychotherapy motivation.

Abbreviations: AdjD, adjustment disorder; GAD, generalized anxiety disorder; PD, panic disorder; PD/AG, panic disorder with agoraphobia; SAD, social anxiety disorder; SP, specific phobia; WAI, Working Alliance Inventory.

^1^Mean (SD); *n* (%).

### 3.3. Symptom Trajectory Classes and Treatment Outcomes

**Figure 2:**
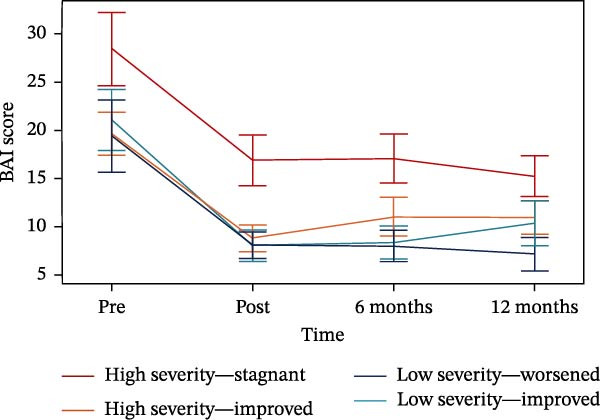
Symptom scores of the latent trajectory classes from pretreatment to follow‐up.

In a final step, we investigated whether the classes differed in their anxiety scores at posttreatment and follow‐up (see Figure 2 and Table 3 for BAI and HAMA scores). While all classes showed improvements, the high severity—stagnant group had higher scores in HAMA at posttreatment (high severity—stagnant vs. high severity—improved: *p* = 0.216, high severity—stagnant vs. low severity—worsened: *p* = 0.016, low severity—worsened vs. high severity—improved: *p* = 0.003). There were no significant differences between the other classes (all *ps* > 0.05). The mixed‐effects model run for BAI scores revealed similar results with the high severity—stagnant group reporting higher scores at posttreatment and 6‐month follow‐up compared to all other trajectory classes (see Supporting Information 2: Table S4 for detailed results). The other three groups did not show any significant differences. However, the high‐severity—stagnant group already showed markedly higher symptoms compared to the other three groups at pretreatment (see Figure 2). At the 12‐month follow‐up, patients in the high severity—stagnant class had improved slightly more, bringing the four classes closer together. Concerning reliable change, the high‐severity—stagnant class showed the lowest proportion of patients being classed as (nonreliably) recovered (see Table 2).

**Table 3:** Treatment outcomes by symptom trajectory class.

Characteristic	High severity—stagnant, *N* = 12^1^	High severity—improved, *N* = 19^1^	Low severity—worsened, *N* = 12^1^	Low severity—improved, *N* = 21^1^
BAI pre	28 (13.03)	20 (9.44)	19 (11.81)	21 (11.38)
BAI post	17 (9.09)	9 (5.44)	8 (4.48)	8 (7.25)
BAI 6MFU	17 (8.81)	11 (8.75)	8 (5.35)	8 (7.20)
BAI 12MFU	15 (7.25)	11 (7.51)	7 (5.74)	10 (9.83)
BAI pre–post RCI				
Deteriorated	0 (0.00%)	0 (0.00%)	0 (0.00%)	0 (0.00%)
Unchanged	7 (58.33%)	8 (53.33%)	2 (22.22%)	3 (25.00%)
Improved	2 (16.67%)	0 (0.00%)	1 (11.11%)	0 (0.00%)
Nonreliably recovered	2 (16.67%)	3 (20.00%)	3 (33.33%)	8 (66.67%)
Recovered	1 (8.33%)	4 (26.67%)	3 (33.33%)	1 (8.33%)
HAMA pre	23 (9.57)	16 (8.14)	16 (7.65)	21 (7.55)
HAMA post	15 (7.62)	11 (6.16)	8 (3.22)	8 (3.98)
BDI pre	25 (9.69)	16 (11.15)	14 (11.10)	11 (7.26)
BDI post	15 (11.13)	9 (9.78)	11 (7.88)	7 (5.02)

Abbreviations: 6MFU, 6‐month follow‐up; 12MFU, 12‐month follow‐up; BAI, Beck Anxiety Inventory; BDI, Beck Depression Inventory; HAMA, Hamilton Anxiety Scale; RCI, reliable change index.

^1^Mean (SD); *n* (%).

## 4. Discussion

Treatment effects vary, and not all patients benefit from treatment to the same extent. Our results provide insights into interindividual differences in symptom progression during transdiagnostic CBT and their relevance for treatment success. We identified four distinct classes of symptom trajectories during treatment with the UP: two groups with initially high anxiety severity, of which one stagnated and one showed improvement, and two groups with initially lower anxiety severity, of which one group worsened and one group showed improvement. The low initial scores of the latter groups were somewhat surprising given that all participants had been diagnosed with a current anxiety disorder in a face‐to‐face clinical interview. In some cases, patients may have lacked awareness to accurately self‐report the severity of their symptoms, for example, not realizing the extent of their avoidance. The observed worsening of symptoms in one class may partly reflect increasing awareness over the course of treatment. Encouragingly, the two improved classes represented the largest proportion of patients, with about 60% of patients showing improvements across the two groups. Corroborating these trajectories with treatment outcome at posttreatment and 6‐month as well as 12‐month follow‐up, we found that, compared to the high severity—stagnant class, the other three classes showed significantly lower anxiety symptoms (in self‐ and clinician report). However, significant differences disappeared in the long‐term follow‐up over 12 months: Independent of class membership, treatment gains were maintained or even improved. From a clinical perspective, this is encouraging as it implies that even patients not showing favorable trajectories during treatment may experience delayed improvement.

In terms of reliable improvement, we found that about half of patients in both high severity classes, the improved as well as stagnant one, were unchanged in the BAI at posttreatment. The low severity‐improved group showed the highest rates of reliable improvement/recovery (75%). However, closer inspection revealed that the majority of these patients were actually nonreliably recovered, given that many patients in this class were below the clinical range of the BAI to begin with. All patients in our sample were diagnosed with an anxiety disorder through an established clinical interview. The BAI has been criticized for its focus on somatic aspects, which may neglect other facets of anxiety, such as emotional aspects. Given the transdiagnostic sample and the prevalence of SAD in our sample, the BAI may not have been the best fit to capture anxiety in our sample. It is also plausible to assume that, given the UP’s focus on introspection of emotions, the treatment itself contributed to making patients more aware of their (somatic) anxiety symptoms, resulting in high BAI ratings.

Patients in our sample differed in the number of sessions they received. We have opted to compare classes with 16 sessions as most patients received this dosage. Since patients who completed more sessions may show delayed improvement of symptoms, we acknowledge that this approach comes with the risk of misclassifying these cases. We thus conducted sensitivity analysis to investigate the stability of class membership: actually, patients who received more than 16 sessions showed small improvements after 16 sessions (1–2 points, see also Supporting Information 2: Figure S3 for individual trajectories), but allocated class memberships did not change when considering longer treatments. How many sessions are “enough” is an ongoing debate in psychotherapy research [41, 42]. Inspection of the trajectories shows that the improved classes show turning points early on and do not experience substantial gains after session 10. We have provided the modules of the UP in standard order, but a personalized order of modules (e.g., earlier exposure) may accelerate change [43]. Personalized treatment termination is also an avenue to consider and may result in closing treatment gaps [44].

Given that the classes started with different intercepts, we explored how baseline and early treatment variables characterize different classes. Overall, we found very comparable demographic and clinical profiles for the different groups. One exception was that the patients initiating treatment with high symptom severity and showing stagnant courses over therapy had a higher number of comorbidities in comparison to those in the low severity‐improved class. This may indicate that patients with more comorbid complaints are more difficult to treat. However, inspection of BAI values over time shows that this group did indeed show a very comparable change pattern to that of the other groups. A systematic review on symptom trajectories in PTSD also revealed that comorbid depression, anxiety, and alcohol abuse emerged as the strongest predictors of poor response [19]. Given the UP’s transdiagnostic focus and comprehensive approach toward comorbidities, a comparison of class trajectories over the course of the UP with other treatments may be informative to gain further insights into how comorbidity impacts trajectories between treatments with a transdiagnostic vs. other approach. The sociodemographic and clinical variables represent fixed and unchangeable factors. However, there may also be factors that are malleable to change and thus constitute a treatment target. Our descriptive exploration of such early treatment variables revealed no apparent differences between groups regarding their coping, hope, expectations, or working alliances.

We have looked at the trajectory of overall anxiety symptoms over the course of treatment. However, it may be worthwhile to nuance this more as certain symptoms of anxiety and specific anxiety disorder symptoms may be more inert and change at a slower rate than others. Longitudinal network models that explore dynamic associations between symptoms of anxiety as well as further transdiagnostic symptoms over time may be an interesting next step to reflect the complexity of the therapeutic process more accurately [45].

One study investigated the trajectories of dropouts in the UP. While this is a different scope, Bentley et al. [46] provided some session‐by‐session means in the OASIS between patients who did and did not drop out of treatment. In their study, completers of the UP dropped from almost 10 to below 6 points on the OASIS by session 16 (data for noncompleters was only available for the beginning of treatment). In contrast, in our study, participants showed lower symptom severity to begin with and a less pronounced pre–post difference. This underlines the necessity to disaggregate average treatment effects and instead investigate interindividual differences. Dropout in our study was low (10%), and the majority of patients completed treatment. This may indicate that patients were retained in treatment even if individuals showed different gains during treatment.

Our investigation is not without limitations. The sample size of 64 patients with anxiety disorders for this psychotherapy study was on the small side for conducting trajectory analyses, which is why we opted for strict LCGA over GMM. Recruiting larger samples remains an inherent challenge in face‐to‐face psychotherapy trials given the resource‐intensive nature of delivering and monitoring multisession treatment in clinical populations. Data collection for this study took place during the COVID‐19 pandemic, which impeded recruitment. However, the 16 within‐person assessments per patient (yielding more than 1000 data points across the sample) and the low dropout rate during treatment strengthen the precision of our individual‐level trajectory estimates and constitute a valuable contribution to gain insights into trajectories during transdiagnostic treatment. Nonetheless, the findings should be interpreted with caution given the limited number of patients. This is particularly relevant for our smallest class (*n* = 12): simulation studies on GMM have shown that classes of around 50 individuals can be recovered with adequate accuracy, but markedly smaller classes showed substantially reduced parameter accuracy compared to recommended benchmarks [47]. We therefore interpret the characteristics of this smallest class with particular caution. For instance, the sample size resulted in us refraining from exploring additional classes and from formal testing of a broad set of predictors of class membership. We dropped one class because we wanted to balance the number of emerging classes with their clinical interpretability. Descriptively, we found some indications of very comparable baseline and early treatment variables, except for male gender, education, and higher comorbidities, but this is not confirmatory and warrants replication. In general, investigations of trajectories are data‐driven rather than hypothesis‐driven. While we were careful in our choices and strove for transparency with sharing data and code on the Open Science Framework (OSF), certain decisions like choosing a cut‐off of 16 sessions are debatable. A consensus on how to investigate trajectories in psychotherapy and how to deal with differing numbers of sessions is currently lacking. Relatedly, this secondary analysis was not preregistered. Preregistering our analytic decisions, such as the session cut‐off, would have further strengthened confidence in these choices. We recommend this for future trajectory studies. Our sample was also rather homogenous and represented an overall WEIRD population, limiting the generalizability of our findings.

## 5. Conclusions

The present findings contribute to the growing literature on interindividual differences in symptom trajectories across psychotherapeutic treatments. During transdiagnostic CBT, latent classes of symptom change can be distinguished and point to malleable factors already present at pretreatment. We found that higher symptom intercepts and a greater number of comorbidities at the onset of therapy may be associated with less favorable courses over time. Closer monitoring and adaptive treatment strategies for this population should be explored.

## Author Contributions

Conceptualization: Birgit Kleim and Isaac Galatzer‐Levy. Data curation: Dominique Recher, Miriam Müller‐Bardorff, Laura E. Meine, and Carmen Schaeuffele. Formal analysis, visualization, writing – original draft: Laura E. Meine and Carmen Schaeuffele. Funding acquisition, project administration, resources, supervision: Birgit Kleim. Investigation: Dominique Recher, Miriam Müller‐Bardorff, Christina Paersch, Ava Schulz, and Birgit Kleim. Methodology: Laura E. Meine, Carmen Schaeuffele, and Birgit Kleim. Software: n/a. Validation: Laura E. Meine, Carmen Schaeuffele, and Dominique Recher. Writing – review and editing: Laura E. Meine, Carmen Schaeuffele, Dominique Recher, Miriam Müller‐Bardorff, Christina Paersch, Ava Schulz, Isaac Galatzer‐Levy, and Birgit Kleim.

## Funding

This work was supported by the Swiss National Science Foundation (Grant 10001C_169827). Open access publishing facilitated by Universitat Zurich, as part of the Wiley ‐ Universitat Zurich agreement via the Consortium of Swiss Academic Libraries.

## Disclosure

The authors provide data and code to follow along on the Open Science Framework, https://doi.org/10.17605/OSF.IO/4M7CU. The funder had no role in study design, data collection and analysis, interpretation of the data and decision to submit the article for publication.

## Ethics Statement

The study was reviewed and approved by the cantonal ethics committee of Zurich (BASEC Number 2017–01443). Written informed consent was obtained from participants.

## Conflicts of Interest

The authors declare no conflicts of interest.

## Supporting Information

Additional supporting information can be found online in the Supporting Information section.

## Supporting information


**Supporting Information 1** STROBE checklist.

**Supporting Information 2** This supporting information provides additional information and data related to the study presented in the main manuscript. It includes supporting tables with additional results, supporting figures with additional descriptives, additional results, and sensitivity analysis results. This supplementary material is essential for understanding the reported results in more detail, providing further evidence for the findings and supporting our conclusions.

## Data Availability

We provide data and code to follow along on OSF: https://doi.org/10.17605/OSF.IO/4M7CU. Further enquiries can be directed to the corresponding authors.
