# Supplementary material for: Symptom Trajectories in Patients With Anxiety Disorders During Transdiagnostic Cognitive Behavioral Therapy
Source: Depress Anxiety. 2026 Jul 21;2026:2743962. doi: 10.1155/da/2743962 (PMC13387043; doi:10.1155/da/2743962)
Supplement: Supplementary file 2 — Supporting Information 2 This supporting information provides additional information and data related to the study presented in the main manuscript. It includes supporting tables with additional results, supporting figures with additional descriptives, additional results, and sensitivity analysis results. This supplementary material is essential for understanding the reported results in more detail, providing further evidence for the findings and supporting our conclusions. [file DA-2026-2743962-s001.pdf]

**Supplementary Materials for**

# Symptom Trajectories in Patients With Anxiety Disorders During Transdiagnostic Cognitive Behavioral Therapy

**Table of Contents**

Supplementary Table 1. Class percentages for the one- to five-class model ................................ 3

Supplementary Table 2. Average latent class probabilities ........................................................ 3

Supplementary Table 3. Results for the four-class solution based on data from 18 sessions......... 4

Supplementary Table 4. Pairwise comparisons of symptom severity between all trajectory classes at all time points ........................................................................................................................ 4

Supplementary Table 5. Reliable change index for clinician-rated anxiety (HAMA) .................. 7

Supplementary Table 6. Reliable change index for BAI and HAMA without norm values .......... 7

Supplementary Table 7. Multinomial logistic regression results for prediction of latent class membership by baseline characteristics .......................................................................... 9

Supplementary Figure 1. Completed therapy sessions by patient .............................................. 10

Supplementary Figure 2. Symptom trajectory classes in the five-class model............................ 11

Supplementary Figure 3. Individual trajectories overlaid with average trajectory by class ......... 12

Supplementary Figure 4. Class trajectories according to the four-class model based on data from 18 sessions ........................................................................................................................ 13

Supplementary Figure 5. Jacobson-Truax plot for pre-post BAI RCI without norm values ........ 14

Supplementary Figure 6. Jacobson-Truax plot for pre-post BAI RCI with norm values ............. 14

Supplementary Figure 7. Jacobson-Truax plot for pre-post HAMA RCI without norm values... 15

Supplementary Figure 8. Jacobson-Truax plot for pre-post HAMA RCI with norm values ........ 15

References for the Supplementary Materials ........................................................................ 16

**Supplementary Table 1. Class percentages for the one- to five-class model**

| Classes | %class1 | %class2 | %class3 | %class4 | %class5 | # participants in smallest class |
|---|---|---|---|---|---|---|
| 1 | 100.00 | | | | | 64 |
| 2 | 59.38 | 40.62 | | | | 26 |
| 3 | 32.81 | 43.75 | 23.44 | | | 15 |
| 4 | 29.69 | 18.75 | 18.75 | 32.81 | | 12 |
| 5 | 26.56 | 20.31 | 20.31 | 15.62 | 17.19 | 10 |

**Supplementary Table 2. Average latent class probabilities**

| Class | mean probability class 1 | mean probability class 2 | mean probability class 3 | mean probabilit y class 4 |
|---|---|---|---|---|
| 1 (high severity - improved) | **0.93** | 0.01 | 0.06 | 0.00 |
| 2 (high severity - stagnant) | 0.03 | **0.97** | 0.00 | 0.00 |
| 3 (low severity - worsened) | 0.06 | 0.00 | **0.93** | 0.00 |
| 4 (low severity - improved) | 0.01 | 0.00 | 0.02 | **0.98** |

**Supplementary Table 3. Results for the four-class solution based on data from 18 sessions**

| | Class | *B* | *SE* | *p* |
|---|---|---|---|---|
| **Intercept** | high severity - stagnant | 12.29 | 0.38 | **< .001** |
| | high severity - improved | 11.06 | 0.41 | **< .001** |
| | low severity - worsened | 6.85 | 0.47 | **< .001** |
| | low severity - improved | 5.64 | 0.45 | **< .001** |
| **Slope** | high severity - stagnant | -0.01 | 0.04 | 0.685 |
| | high severity - improved | -0.32 | 0.04 | **< .001** |
| | low severity - worsened | 0.17 | 0.05 | **< .001** |
| | low severity - improved | -0.17 | 0.04 | **< .001** |

**Supplementary Table 4. Pairwise comparisons of symptom severity between all trajectory classes at all time points**

| Contrast | *Estimate* | *SE* | *t* | *p* |
|---|---|---|---|---|
| **pre-treatment** | | | | |
| (high severity - stagnant) - (high severity - improved) | 8.80 | 3.16 | 2.78 | **0.031** |
| (high severity - stagnant) - (low severity - worsened) | 8.93 | 3.58 | 2.49 | 0.066 |
| (high severity - stagnant) - (low severity - improved) | 6.74 | 3.30 | 2.05 | 0.176 |
| (high severity - improved) - (low severity - worsened) | 0.13 | 3.28 | 0.04 | 1.000 |
| (high severity - improved) - (low severity - improved) | -2.06 | 2.96 | -0.70 | 0.899 |

| | | | | |
|---|---|---|---|---|
| (low severity - worsened) - (low severity - improved) | -2.19 | 3.41 | -0.64 | 0.918 |
| **post-treatment** | | | | |
| (high severity - stagnant) - (high severity - improved) | 8.15 | 3.21 | 2.54 | 0.059 |
| (high severity - stagnant) - (low severity - worsened) | 9.13 | 3.54 | 2.58 | 0.053 |
| (high severity - stagnant) - (low severity - improved) | 8.64 | 3.10 | 2.79 | **0.031** |
| (high severity - improved) - (low severity - worsened) | 0.98 | 3.28 | 0.30 | 0.991 |
| (high severity - improved) - (low severity - improved) | 0.50 | 2.80 | 0.18 | 0.998 |
| (low severity - worsened) - (low severity - improved) | -0.48 | 3.17 | -0.15 | 0.999 |
| **6-months follow-up** | | | | |
| (high severity - stagnant) - (high severity - improved) | 6.03 | 3.14 | 1.92 | 0.225 |
| (high severity - stagnant) - (low severity - worsened) | 9.39 | 3.54 | 2.65 | **0.044** |
| (high severity - stagnant) - (low severity - improved) | 8.47 | 3.15 | 2.69 | **0.040** |
| (high severity - improved) - (low severity - worsened) | 3.36 | 3.21 | 1.05 | 0.723 |
| (high severity - improved) - (low severity - improved) | 2.44 | 2.77 | 0.88 | 0.815 |
| (low severity - worsened) - (low severity - improved) | -0.91 | 3.21 | -0.28 | 0.992 |
| **12-months follow-up** | | | | |

| | | | | |
|---|---|---|---|---|
| (high severity - stagnant) - (high severity - improved) | 4.30 | 3.14 | 1.37 | 0.521 |
| (high severity - stagnant) - (low severity - worsened) | 8.37 | 3.54 | 2.37 | 0.089 |
| (high severity - stagnant) - (low severity - improved) | 5.07 | 3.15 | 1.61 | 0.377 |
| (high severity - improved) - (low severity - worsened) | 4.07 | 3.21 | 1.27 | 0.585 |
| (high severity - improved) - (low severity - improved) | 0.76 | 2.77 | 0.28 | 0.993 |
| (low severity - worsened) - (low severity - improved) | -3.30 | 3.21 | -1.03 | 0.733 |

*Note*. Symptom severity was assessed with the Beck Anxiety Inventory (BAI). Significant *p*-values ($p < 0.05$) were bolded.

**Supplementary Table 5. Reliable change index for clinician-rated anxiety (HAMA)**

| **Characteristic** | **high severity - stagnant**, *N* = 12[1] | **high severity - improved**, *N* = 19[1] | **low severity - worsened**, *N* = 12[1] | **low severity - improved**, *N* = 21[1] |
|---|---|---|---|---|
| HAMA pre-post RCI | | | | |
| deteriorated | 1 (8.33%) | 2 (13.33%) | 1 (10.00%) | 0 (0.00%) |
| unchanged | 1 (8.33%) | 1 (6.67%) | 0 (0.00%) | 0 (0.00%) |
| improved | 4 (33.33%) | 1 (6.67%) | 0 (0.00%) | 0 (0.00%) |
| non reliably recovered | 1 (8.33%) | 6 (40.00%) | 1 (10.00%) | 4 (22.22%) |
| recovered | 5 (41.67%) | 5 (33.33%) | 8 (80.00%) | 14 (77.78%) |

*Note*. No norm data was available for the HAMA, but the instruction to the German HAMA contains means and SDs for different anxiety populations assessed with the English-speaking HAMA. To compare categories with the Beck Anxiety Inventory (BAI), we opted to calculate the RCI using means and SDs from GAD patients, as reported in Matza et al. (2010). HAMA = Hamilton Anxiety Scale; RCI = reliable change index
[1] n (%)

**Supplementary Table 6. Reliable change index for BAI and HAMA without norm values**

| **Characteristic** | **high severity - stagnant**, *N* = 12[1] | **high severity - improved**, *N* = 19[1] | **low severity - worsened**, *N* = 12[1] | **low severity - improved**, *N* = 21[1] |
|---|---|---|---|---|
| BAI pre-post RCI | | | | |
| deteriorated | 0 (0.00%) | 0 (0.00%) | 0 (0.00%) | 0 (0.00%) |
| unchanged | 9 (75.00%) | 11 (73.33%) | 5 (55.56%) | 11 (91.67%) |

| | | | | |
|---|---|---|---|---|
| improved | 3 (25.00%) | 4 (26.67%) | 4 (44.44%) | 1 (8.33%) |
| non reliably recovered | 0 (0.00%) | 0 (0.00%) | 0 (0.00%) | 0 (0.00%) |
| recovered | 0 (0.00%) | 0 (0.00%) | 0 (0.00%) | 0 (0.00%) |
| HAMA pre-post RCI | | | | |
| deteriorated | 1 (8.33%) | 2 (13.33%) | 1 (10.00%) | 0 (0.00%) |
| unchanged | 2 (16.67%) | 7 (46.67%) | 1 (10.00%) | 4 (22.22%) |
| improved | 9 (75.00%) | 6 (40.00%) | 8 (80.00%) | 14 (77.78%) |
| non reliably recovered | 0 (0.00%) | 0 (0.00%) | 0 (0.00%) | 0 (0.00%) |
| recovered | 0 (0.00%) | 0 (0.00%) | 0 (0.00%) | 0 (0.00%) |

*Note*. BAI = Beck Anxiety Inventory; RCI = reliable change index; HAMA = Hamilton Anxiety Scale

[1] n (%)

**Supplementary Table 7. Multinomial logistic regression results for prediction of latent class membership by baseline characteristics**

| | high severity - stagnant vs high severity - improved | | | high severity - stagnant vs low severity - improved | | | high severity - stagnant vs low severity - worsened | | |
|---|---|---|---|---|---|---|---|---|---|
| **Variable** | **OR[1]** | **95% CI[1]** | ***p*** | **OR[1]** | **95% CI[1]** | ***p*** | **OR[1]** | **95% CI[1]** | ***p*** |
| Sex (male) | 0.19 | 0.03, 1.26 | 0.086 | 0.68 | 0.13, 3.55 | 0.645 | 0.12 | 0.01, 1.46 | 0.096 |
| Education (higher) | 1.80 | 0.34, 9.51 | 0.487 | 1.04 | 0.20, 5.53 | 0.965 | 2.12 | 0.32, 13.9 | 0.433 |
| # comorbidities | 0.66 | 0.34, 1.30 | 0.230 | 0.50 | 0.26, 0.98 | **0.045** | 0.43 | 0.18, 1.01 | 0.053 |
| Medication status (yes) | 0.30 | 0.05, 1.66 | 0.168 | 0.32 | 0.06, 1.70 | 0.183 | 0.38 | 0.06, 2.51 | 0.314 |

[1] OR = Odds Ratio, CI = Confidence Interval; significant p-values ($p < 0.05$) were bolded.

**Supplementary Figure 1. Completed therapy sessions by patient**

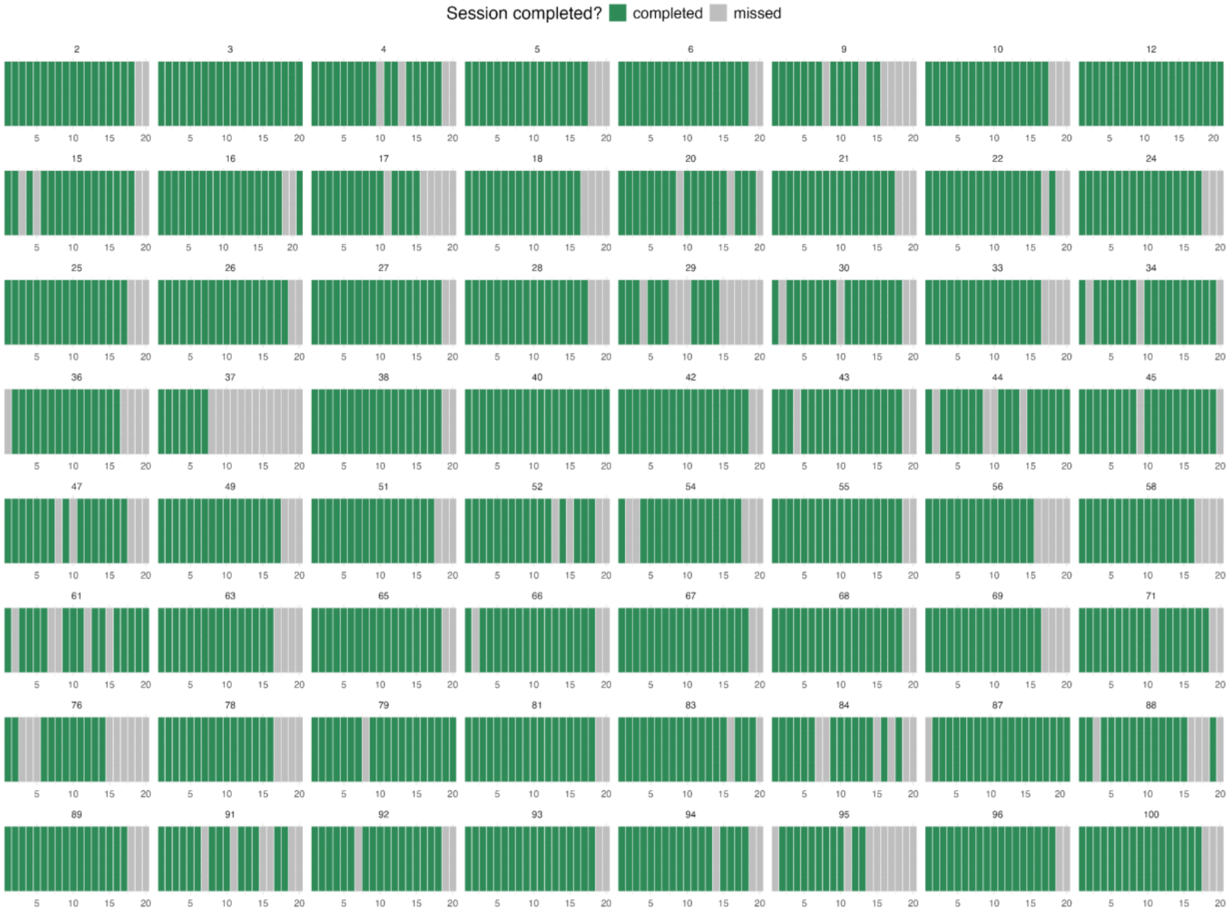


*Note.* Each panel shows completed and missed symptom reports per session for one patient (numbers are anonymized IDs). We display up to 21 sessions; however, the maximum number of sessions required up to conclusion of treatment varied between patients, with fewer sessions needed for most.

**Supplementary Figure 2. Symptom trajectory classes in the five-class model**

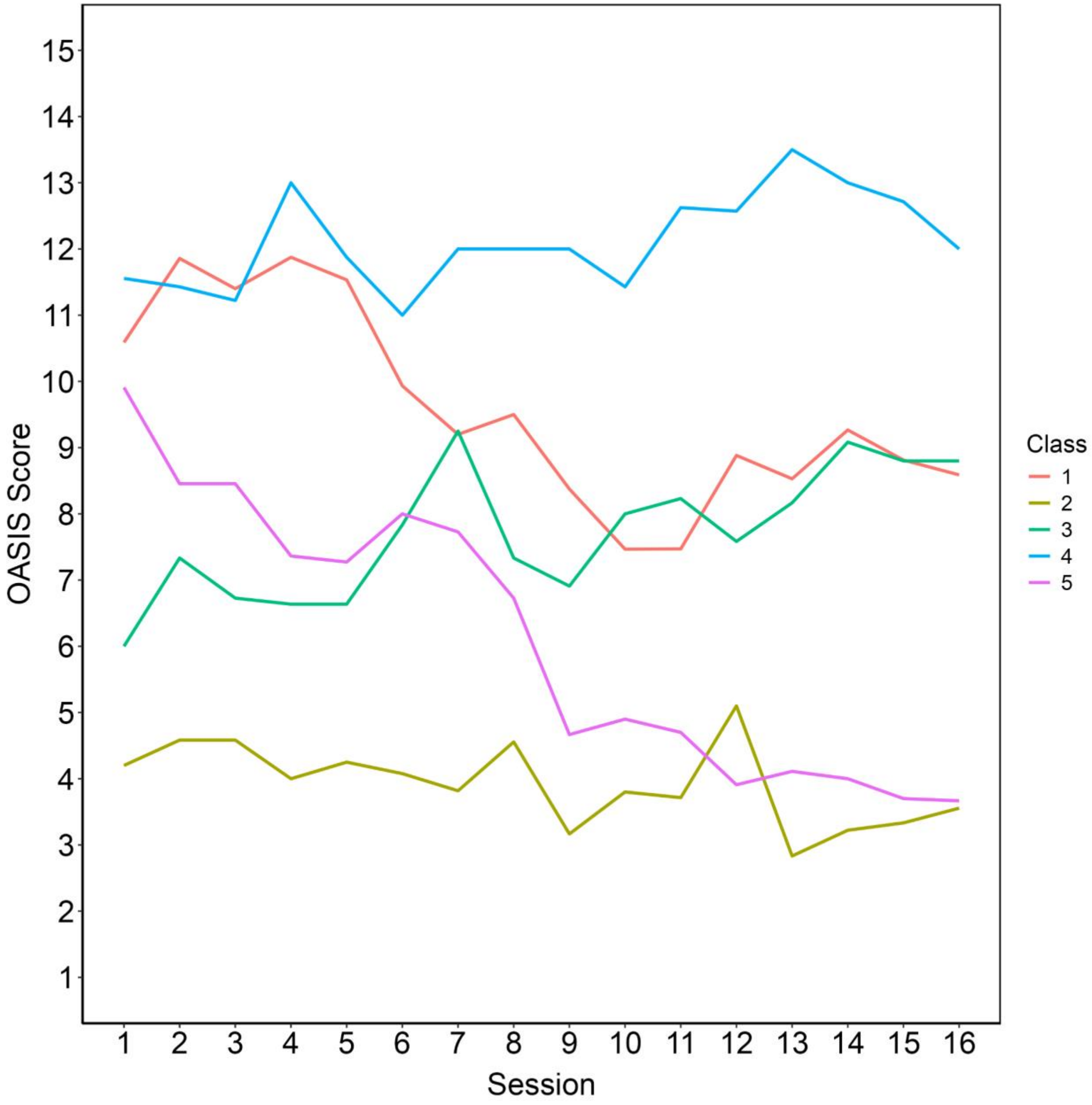

**Supplementary Figure 3. Individual trajectories overlaid with average trajectory by class**

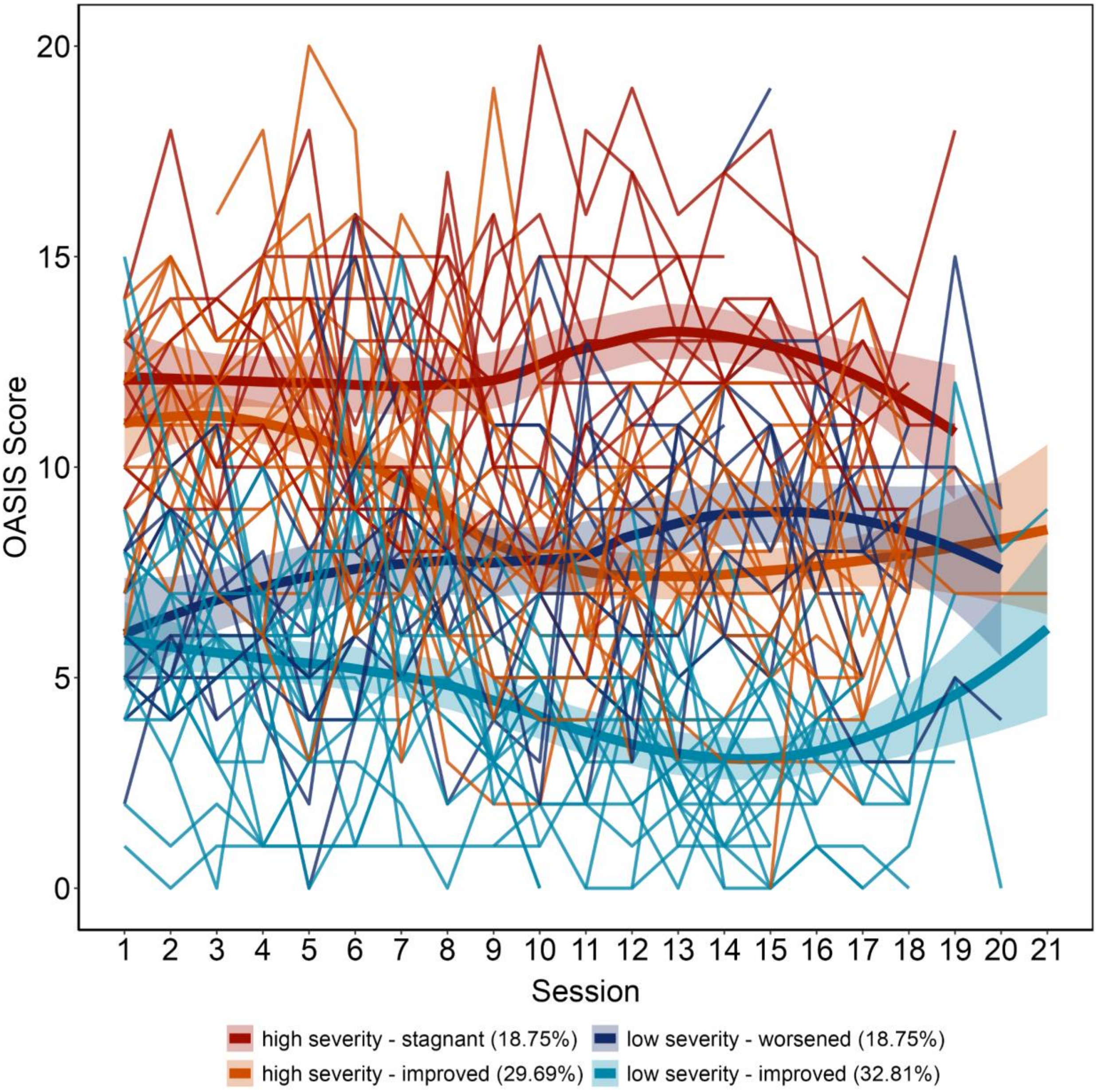


*Note.* While the LCGA was based on data from 16 sessions, here, we show data from all available sessions.

**Supplementary Figure 4. Class trajectories according to the four-class model based on data from 18 sessions**

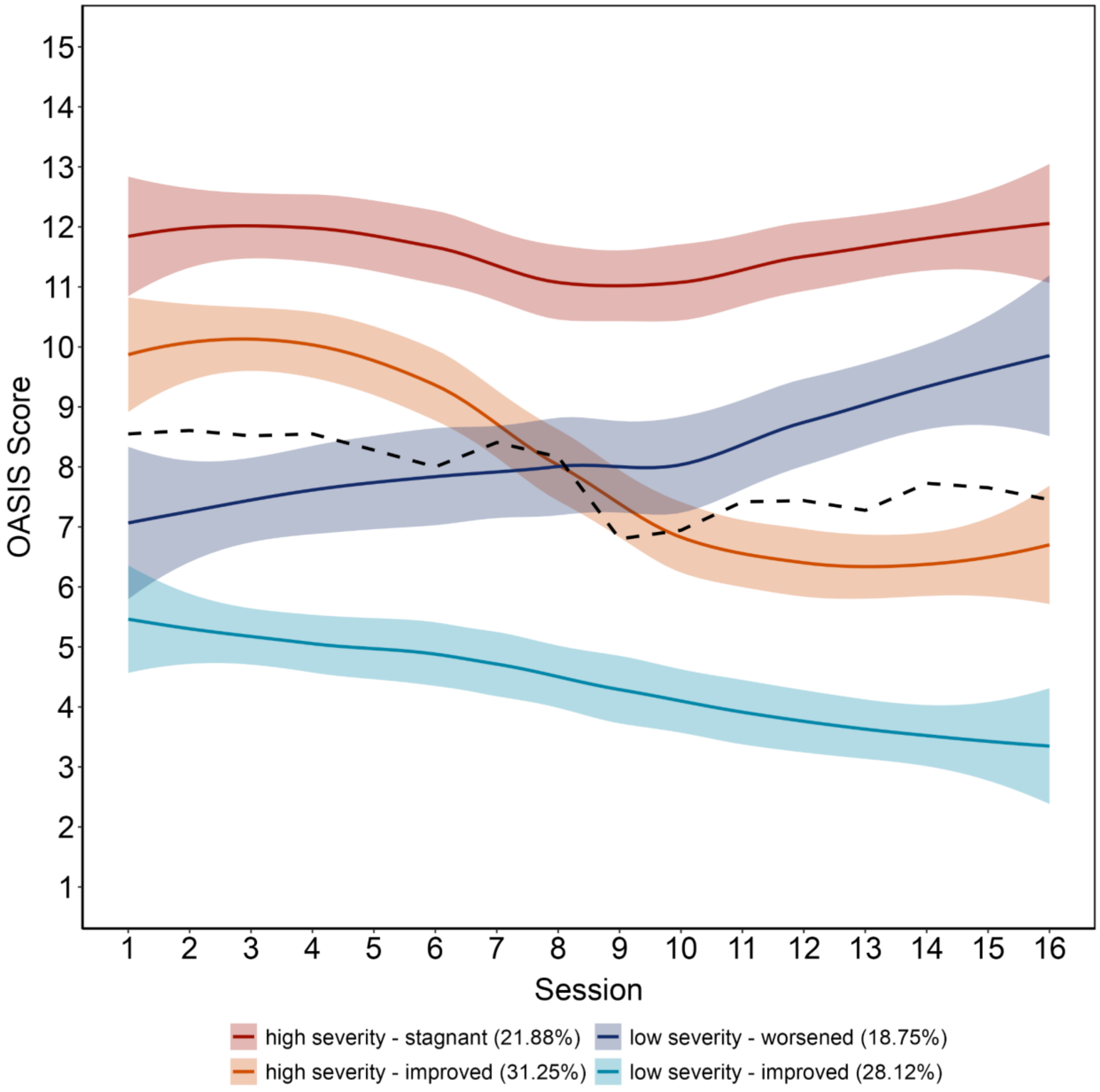

**Supplementary Figure 5. Jacobson-Truax plot for pre-post BAI RCI without norm values**

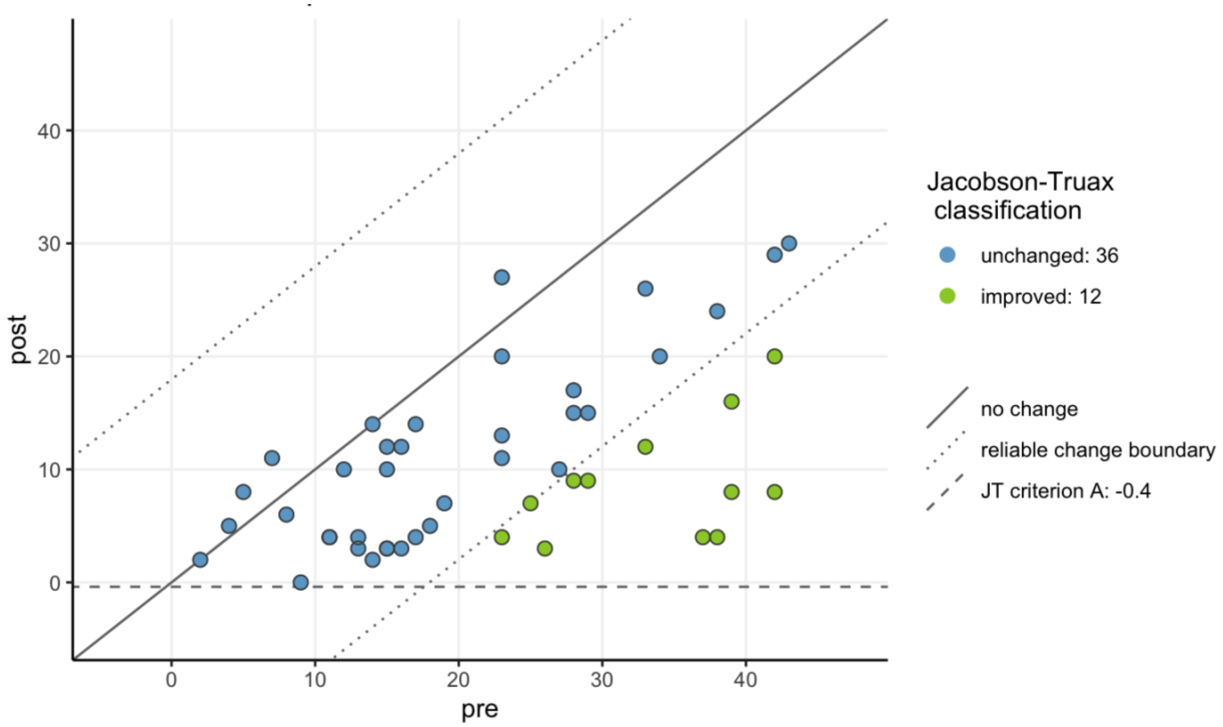


*Note.* BAI = Beck Anxiety Inventory; RCI = reliable change index

**Supplementary Figure 6. Jacobson-Truax plot for pre-post BAI RCI with norm values**

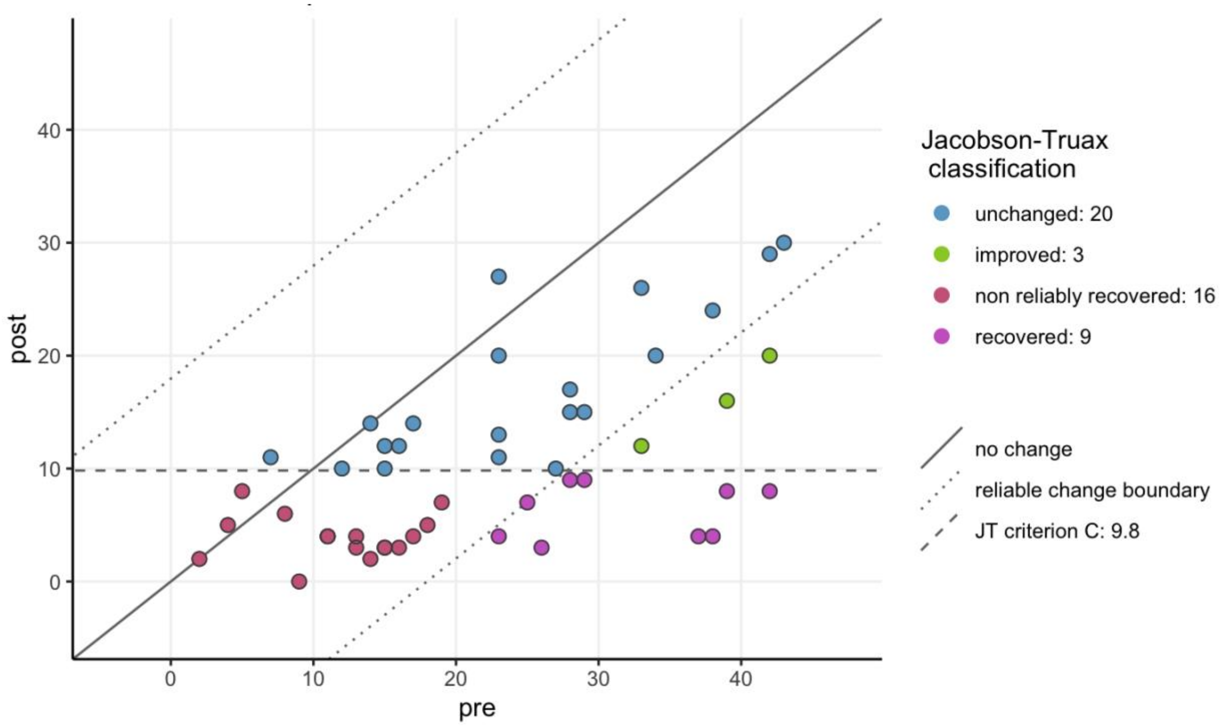

*Note.* BAI = Beck Anxiety Inventory; RCI = reliable change index

**Supplementary Figure 7. Jacobson-Truax plot for pre-post HAMA RCI without norm values**

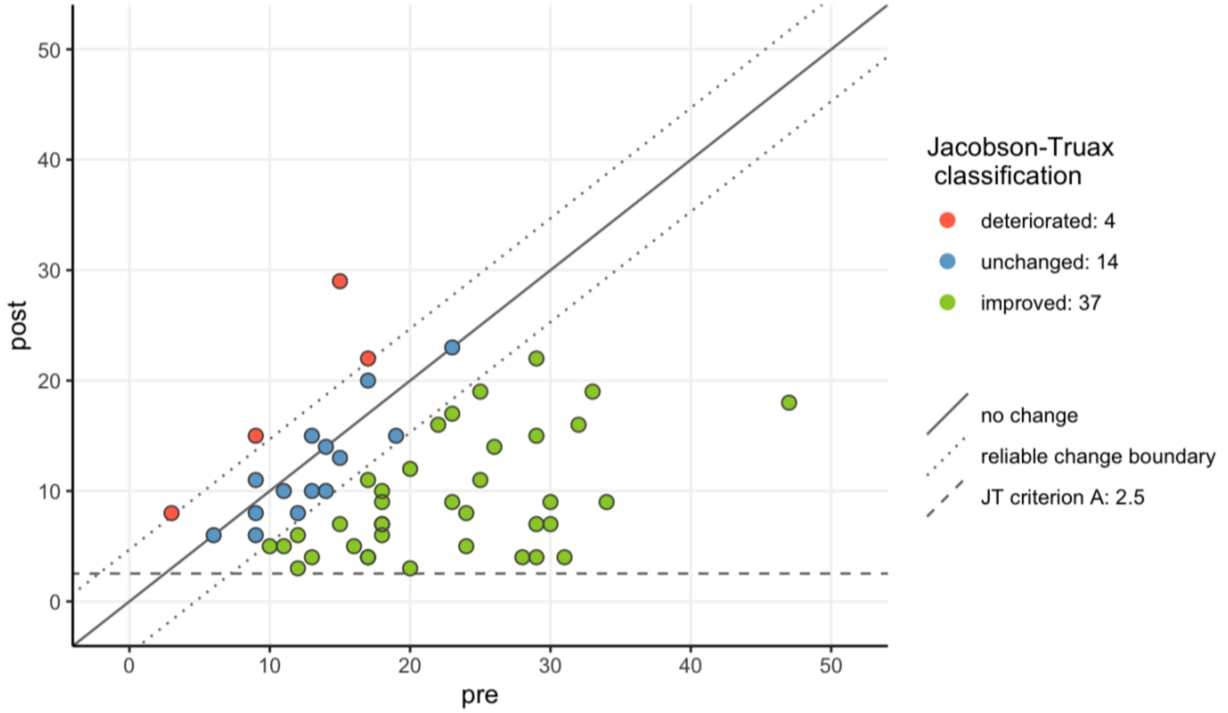


*Note.* HAMA = Hamilton Anxiety Scale; RCI = reliable change index

**Supplementary Figure 8. Jacobson-Truax plot for pre-post HAMA RCI with norm values**

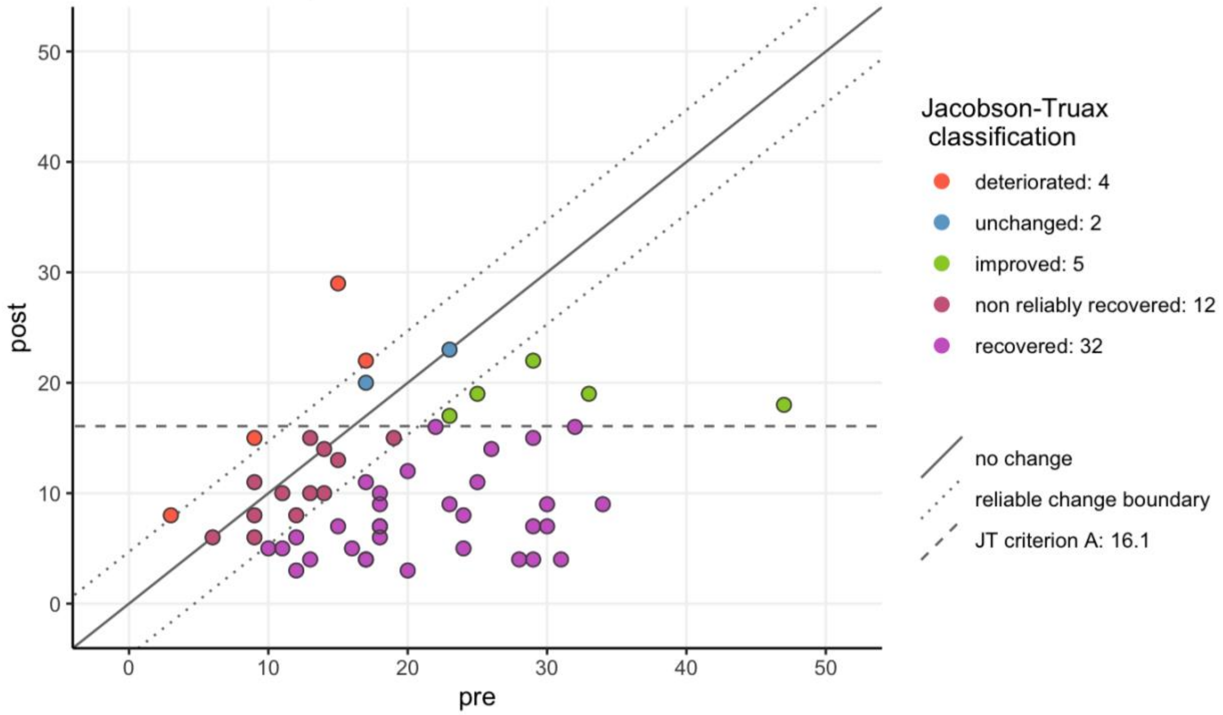


*Note.* HAMA = Hamilton Anxiety Scale; RCI = reliable change index

**References for the Supplementary Materials**

Matza, L. S., Morlock, R., Sexton, C., Malley, K., & Feltner, D. (2010). Identifying HAM-A cutoffs for mild, moderate, and severe generalized anxiety disorder. *International journal of methods in psychiatric research*, *19*(4), 223-232.
